# Assessment and *In Vivo* Scoring of Murine Experimental Autoimmune Uveoretinitis Using Optical Coherence Tomography

**DOI:** 10.1371/journal.pone.0063002

**Published:** 2013-05-14

**Authors:** Colin J. Chu, Philipp Herrmann, Livia S. Carvalho, Sidath E. Liyanage, James W. B. Bainbridge, Robin R. Ali, Andrew D. Dick, Ulrich F. O. Luhmann

**Affiliations:** 1 Department of Genetics, UCL Institute of Ophthalmology, London, United Kingdom; 2 NIHR Biomedical Research Centre for Ophthalmology, Moorfields Eye Hospital, London, United Kingdom; 3 Unit of Ophthalmology, School of Clinical Sciences, University of Bristol, Bristol, United Kingdom; University Hospital La Paz, Spain

## Abstract

Despite advances in clinical imaging and grading our understanding of retinal immune responses and their morphological correlates in experimental autoimmune uveoretinitis (EAU), has been hindered by the requirement for post-mortem histology. To date, monitoring changes occurring during EAU disease progression and evaluating the effect of therapeutic intervention in real time has not been possible. We wanted to establish whether optical coherence tomography (OCT) could detect intraretinal changes during inflammation and to determine its utility as a tool for accurate scoring of EAU. EAU was induced in *C57BL/6J* mice and animals evaluated after 15, 26, 36 and 60 days. At each time-point, contemporaneous Spectralis-OCT scanning, topical endoscopic fundal imaging (TEFI), fundus fluorescein angiography (FFA) and CD45-immunolabelled histology were performed. OCT features were further characterised on retinal flat-mounts using immunohistochemistry and 3D reconstruction. Optic disc swelling and vitreous opacities detected by OCT corresponded to CD45+ cell infiltration on histology. Vasculitis identified by FFA and OCT matched perivascular myeloid and T-cell infiltrates and could be differentiated from unaffected vessels. Evolution of these changes could be followed over time in the same eye. Retinal folds were visible and found to encapsulate mixed populations of activated myeloid cells, T-cells and microglia. Using these features, an OCT-based EAU scoring system was developed, with significant correlation to validated histological (Pearson r^2^ = 0.6392, P<0.0001, n = 31 eyes) and TEFI based scoring systems (r^2^ = 0.6784, P<0.0001). OCT distinguishes the fundamental features of murine EAU *in vivo*, permits dynamic assessment of intraretinal changes and can be used to score disease severity. As a result, it allows tissue synchronisation with subsequent cellular and functional assessment and greater efficiency of animal usage. By relating OCT signals with immunohistochemistry in EAU, our findings offer the opportunity to inform the interpretation of OCT changes in human uveitis.

## Introduction

As an animal model with features resembling those of human intraocular autoimmune inflammatory disease, murine experimental autoimmune uveoretinitis (EAU) has been central to many of the advances and translational studies in ocular immunology over the last decade [1], [2]. Spontaneous and inducible EAU can be obtained using a variety of wildtype and transgenic mouse strains to achieve different disease kinetics, severity and polarity of immune responses [3]. More recently, laboratories have advanced our knowledge of EAU and thus human disease through assays such as retinal multiparameter flow cytometric analysis. This has facilitated the dissection of molecular mechanisms that determine disease phenotype and the function, for example, of recruited macrophages [4], [5]. To date however, the inability to identify histological changes in real time has limited our ability to correlate cellular infiltrate and function with morphological changes.

Despite increasing sensitivity of flow cytometric analysis within the retina, a robust readout of disease status would improve the utility of EAU for preclinical translational studies. Currently, morphological assessments are only possible with histology and semi-quantitative scoring systems using either haematoxylin/eosin [6] or CD45 (pan-leukocyte) immunolabelling [7]. Whilst histology and flow cytometric assessment of cell infiltrate provide detailed information at a single time-point, the terminal nature of these analyses intrinsically precludes repeated scoring of the same animal. This limitation is compounded by variation in EAU development, amplitude and dynamics, with asymmetry even between the eyes of the same animal [8]. To circumvent this, data from large cohorts of mice are normally pooled, sacrificing information on individual variation and amalgamating results into potentially misleading averages.

Progress towards overcoming these limitations culminated in the introduction of topical endoscopic fundal imaging (TEFI) as a scoring platform for murine EAU [9]. Using this technique, repeated detailed images of the retina from the same eye could be obtained over the entire timecourse of disease. By scoring these fundus photographs for disc, vessel and structural changes, a surrogate measure can be obtained, which has been shown empirically to approximate to infiltration and histological scores [10]. Thereby it has been possible to correlate disease changes with cellular infiltration and confirm the effects of novel treatments e.g. of drugs that affect cell trafficking [11], [12].

Whilst a substantial advance, TEFI requires high levels of light exposure and corneal instrumentation, that with cumulative use may have the potential to cause damage to the retina and ocular surface [13]. Disease can also be overestimated, predominantly by failing to define normal optic disc appearance or in later stages confusing perivascular scarring as active vasculitis. Furthermore TEFI is unable to quantify vitreous infiltration or resolve intraretinal changes and thus remains an inadequate surrogate score for the gold standard of histology (using 2D colour images to approximate 3D intraretinal tissue changes). For these reasons the pursuit of improved systems has continued.

Optical coherence tomography (OCT) has revolutionised the diagnosis, monitoring and treatment of human retinal and macular disease and its application is now widespread in clinical practice [14], [15]. OCT discerns structure in the retina based on the scatter of reflected light and can, dependent on laser wavelength, reach axial resolutions down to 4 µm, advancing the prospect of observing histological detail *in vivo* [16]. Over recent years commercially available systems have been improved, adapted for use in animals and now allow reliable imaging of features in the rodent eye [17]. The most significant benefit of an OCT-based EAU scoring system arises from the near-histological level of detail, which would permit the same accurate staging of disease, but repeated over time in the same eye. As a result, dynamic intraretinal changes can be monitored and better defined *in vivo*.

In this study we aimed to refine our understanding of the correlation between morphological changes and OCT features by assessing the ability of OCT to detect retinal changes in *C57BL/6J* mice undergoing EAU and devising a scoring system to parallel histological scores. We found that OCT could distinguish between diseased and control animals based on good correlation with fundus fluorescein angiography (FFA), TEFI and histology. Critical aspects of disease such as optic disc swelling, vasculitis and structural damage were delineated with OCT and further defined using immunohistochemistry and 3D reconstruction. We were also able to define the nature of a so far ill understood OCT feature, as an unusual retinal vascular malformation that arises from day 36 in EAU. Finally it was possible to construct a scoring system, which we confirmed correlated well with both histology and TEFI based scores. Together this data provides evidence that real time OCT monitoring and quantification of disease progression in murine EAU is feasible and can assist in defining the functional-structural consequences of pathological immune responses in the retina.

## Materials and Methods

### Animals and Induction of EAU

Female *C57BL/6J* mice (Harlan, UK) at seven-weeks of age were immunised for EAU and maintained with food and water *ad libitum* in cages adjacent to complete Freund’s adjuvant (CFA) controls. Mice with vascular abnormalities were genotyped by sequencing and confirmed as *rd8* (*Crb1* ^rd8/rd8^) mutation free [18]. All procedures were performed under the UK Home office project licence PPL 70/1279 and conformed to the Association of Research in Vision and Ophthalmology (ARVO) statement for the use of animals in ophthalmic and vision research.

For EAU induction, 500 µg per mouse of human RBP-3_1–20_ peptide was injected subcutaneously, emulsified in CFA supplemented with 1.5 mg/mL *M. tuberculosis* H37RA (Difco laboratories, BD, Oxford, UK). 1.5 µg of Pertussis toxin was simultaneously administered into the peritoneal space (Tocris Bioscience, Bristol, UK). CFA control mice received the same regime, including pertussis toxin and CFA, but substituting PBS for RBP-3 peptide.

For all *in vivo* procedures, animals underwent anaesthesia with injection of 200 µl medetomidine hydrochloride (1 mg/ml), 100 mg/ml ketamine (Orion pharmaceuticals, Helskinki, Finland), and water in the ratio 5∶3:42. Post-procedure anaesthesia was reversed by injection of an equal volume of AntiSedan (Pfizer pharmaceuticals, USA). Pupils were dilated for 5 minutes with topical tropicamide 1% and phenylephrine 2.5% (Chauvin Pharmaceuticals, Romford, UK).

### Topical Endoscopic Fundus Imaging (TEFI)

Using a published method, a 5 cm endoscope 3 mm in outer diameter (1218 AA; Karl Storz, Tuttlingen, Germany), was connected by fibre-optic cable to a Nikon D300s digital camera with a 12.3 megapixel charge-coupled device sensor and Viscotears (Novartis Pharmaceuticals, UK) was used as a coupling agent for corneal contact [10]. Contrast and brightness of the fundus images were adjusted in Adobe Photoshop CS5.5 (Adobe Systems Incorporatied, San Jose, USA). Using an adapted clinical grading system, fundus images were scored in a masked fashion by three assessors, according to changes in the optic disc, vessels and surrounding retina [9].

### Optical Coherence Tomographic (OCT) Imaging and Fundus Fluorescein Angiography (FFA)

Spectralis™ HRA and OCT (Heidelberg Engineering, Heidelberg, Germany) with an aspheric, 80 mm focal length, near-infra-red lens adaptor (Linos, Qioptiq, Luxembourg) was used to capture images with a 30-degree angle of view. The OCT+IR channel was used to correlate retinal position with the obtained OCT optical section. Animals were injected in the peritoneal space with 200 µl of 2% fluorescein in PBS. Simultaneous OCT and fundus fluorescein angiography was obtained using the autofluorescent channel of the Spectralis™ HRA. Viewing module 5.7.0.9 and acquisition module 5.6.3.0 were used with Heidelberg eye explorer version 1.7.1.0. Images were exported and processed in Adobe Photoshop CS5.5 (Adobe Systems Incorporated, San Jose, USA). Details of OCT-based EAU scoring can be found in the results section and **Figure S1**.

### Immunohistochemistry and Histology based EAU Scoring

For CD45 histological scoring, enucleated eyes were snap frozen in optimal cutting temperature medium, serially sectioned at 15 µm and stained as previously published [10]. Briefly, following acetone fixation and non-specific rabbit serum block, anti-CD45 antibody (AbD Serotec, Kidlington, UK) labelling underwent chromogenic staining using a DAB substrate kit and haematoxylin counterstain (Vector laboratories, Peterborough, UK). Scoring was performed across the whole eye, selecting the most severe example of disease present and marked according to a previously described system capable of detecting subtle changes in infiltration and structure [7].

Retinal flat-mounts were stained by adapting a published protocol [18]. The cornea and lens are removed prior to four relieving incisions and separation of retina from RPE along the sub-retinal space. Following dissection, the retinae were placed into 100% methanol overnight, then blocked and stained in 1% BSA (Sigma Aldrich, UK), 3% Triton X-100 and 5% non-specific goat serum (AbD serotec, UK). Antibodies were all used at 1∶200 dilution including anti-collagen IV (code: 2150-1470, AbD serotec, UK), an equal mix of anti-CD4 (code: 553043, BD Biosciences, UK) and anti-CD8 (code: 553027, BD Biosciences, UK), anti-Iba1 (code: 019-19741, WAKO, Osaka, Japan) and biotinylated lectin B4 from *Bandeiraea simplicifolia* (code: L2140, Sigma aldrich, UK) [19]. Alexa fluor 488, 546 and 633 secondary antibodies (Invitrogen-Molecular Probes, Leiden, The Netherlands) were used at 1∶500 dilution. Images were acquired by confocal laser scanning microscopy (Leica DM5500Q, Leica Microsystems, Germany). High-resolution z-stack images were used for 3D reconstruction using Imaris software (Bitplane, Zurich, Switzerland).

### Statistical Analysis

GraphPad Prism 5 for Windows was employed for statistical analyses (GraphPad Software Inc, La Jolla, USA). A P-value below 0.05 was considered statistically significant.

## Results

### OCT Detects Key Tissue Changes Associated with EAU in C57BL/6 Mice

To determine if EAU-induced changes could be detected by OCT in *C57BL/6* mice and used to score disease status, three independent cohorts totalling 33 mice (12, 12 and 9 respectively) were induced at two-week intervals. Assessment was performed at days 15, 26, 36 and 60 post-induction. CFA control animals, which received only complete Freund’s adjuvant and pertussis toxin, were followed through to day 60. At each time-point all animals underwent TEFI and OCT scanning, with simultaneous fundus fluorescein angiography (FFA) in a subset. Animals were selected randomly at each imaging session for histological or retinal flat-mount analysis (n = 5–7 eyes per group). Other cohorts were observed up to 80 days post-induction. We commenced our study by looking at features critical to histological EAU assessment and wished to determine whether it was possible to detect the same changes by OCT. See **Figure S2** for a description of OCT correlation to retinal anatomy.

### Optic Disc Changes

**Figure 1:**
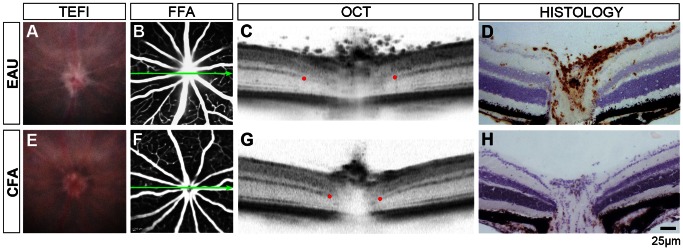
EAU in the *C57BL/6J* mouse induces optic nerve head changes detectable on OCT. Representative images from an eye 15 days post-induction of EAU (**A–D**) and matched CFA control (**E–H**) using four modalities. Blurred optic disc margins on TEFI (**A**) and disc hyperfluorescence on FFA (**B**) are seen in EAU. Green arrows show the location and orientation of the OCT scan. Red dots indicate where the discrete signal from the outer plexiform layer becomes obscured by disc enlargement, as a landmark to compare between eyes. Note a wider separation and vitreous opacities in EAU (**C**). Matched histology demonstrates CD45+ infiltration (DAB in brown with haematoxylin counterstain) during EAU (**D**). With TEFI alone (**E**), it is possible that changes in disc appearance would erroneously be identified as disease with infiltration in the CFA control, as OCT and immunohistochemistry do not corroborate any evidence of disease.

Initial changes in EAU are evident at the optic disc and observed clinically by TEFI as optic nerve swelling with loss of margin definition. An example is shown in **Figure 1A**. In the same representative animal, confirmation of disease activity is demonstrated by FFA as leakage of dye (**Figure 1B**) when compared with the matched CFA control. In EAU, hyperfluorescence at the optic disc and vascular enlargement occur as a result of inflammatory infiltrate and vascular barrier breakdown. Using OCT scanning, a section through the horizontal centre of the disc can be obtained which reflects underlying changes of infiltration and oedema (**Figure 1C**). CD45+ immunohistological labelling from the same eye confirms the presence of immune cells infiltrating the disc (**Figure 1D**), which are absent in the control (**Figure 1H**). Furthermore, any oedema observed by OCT imaging (**Figures 1B & 1C**) is largely lost during tissue processing and cells infiltrating the vitreous are frequently displaced in sectioning. Most importantly, although TEFI in the CFA control animal demonstrates a clinically diseased optic nerve (**Figure 1E**), with FFA, OCT and histology there is no evidence of inflammation. In contrast during EAU, vitreous opacities around the disc are visible on OCT. Both vitreous activity and disc swelling appear to reach a peak between days 26 to 36 post-induction and decrease by day 60 (**Figure S3**). The data shows that OCT delineates, with greater sensitivity and specificity than TEFI, both optic disc and vitreous cavity inflammatory changes throughout EAU; two components central to the assessment of disease status.

### Structural Changes

**Figure 2:**
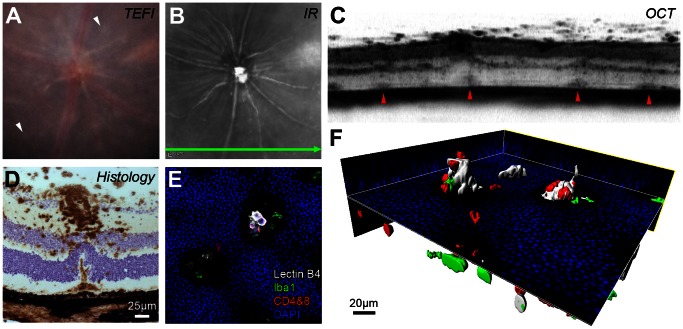
Abnormal OCT signals *in vivo* correspond to structural changes and associated cellular infiltrates. Images from an eye 80 days post-induction of EAU (**A–C**) can be interpreted using matched *ex vivo* histology (**D–F**). On TEFI (**A**) multiple pale retinal lesions (white arrowheads) are present. The infrared (IR) image (**B**) marks the position and orientation of the simultaneously obtained OCT scan (**C**). Focal changes at the level of the subretinal space/deep retina correspond to the observed lesions (red arrowheads). (**D**) Note the CD45+ cellular infiltrate (DAB in brown with haematoxylin counterstain) around the superficial vessel and under the displaced outer nuclear layer. A limited projection image (**E**) and 3D reconstruction (**F**) of the stained retinal flat-mount identifies these lesions as folds in the outer nuclear layer containing T cell - CD4+ & CD8+ (red), myeloid - lectin B4 binding (white) and Iba1+ (green) cell populations protruding from the subretinal space (DAPI in blue). **Movie S1** shows an animated version of this panel.

To assess the ability of OCT to detect intraretinal structural changes, we examined morphological alterations in eyes with widespread, discrete, chorioretinal lesions - a hallmark of disease, which increases in later stages (**Figure 2A** **, white arrows**). OCT scans of these lesions revealed dome-shaped signals at the level corresponding to the subretinal space, which displace the photoreceptor and outer nuclear layer upwards into the inner nuclear layer (**Figure 2B, C**). In many instances, the largest lesions occur in proximity to heavily infiltrated vessels and a representative example is shown with histology (**Figure 2D**). This additionally identifies these lesions as the ‘retinal folds’ frequently observed in histological sections and adopted into the structural assessment of several EAU scoring systems [6], [7]. As these folds are poorly characterised we stained retinal flat-mounts for T-cells, microglia and myeloid cells to assess their spatial relationship (**Figure 2E**). The representative example underwent 3D reconstruction, which illustrates domed cavities projecting into the outer nuclear layer, with a mixed population of cells contained within most likely extending from the subretinal space (**Figure 2F**). Lectin B4, which labels a subset of the myeloid lineage as well as vascular endothelium [20] was used to stain this structure. As no vessels were observed in the regions of the folds, or when stained with collagen IV (data not shown), lectin B4 expression represents activated myeloid cells [19] and 3D reconstruction further emphasises the encapsulation of inflammatory cells (myeloid and T-cells) in the fold and within the subretinal space (see **Movie S1**).

### Vascular Changes

**Figure 3:**
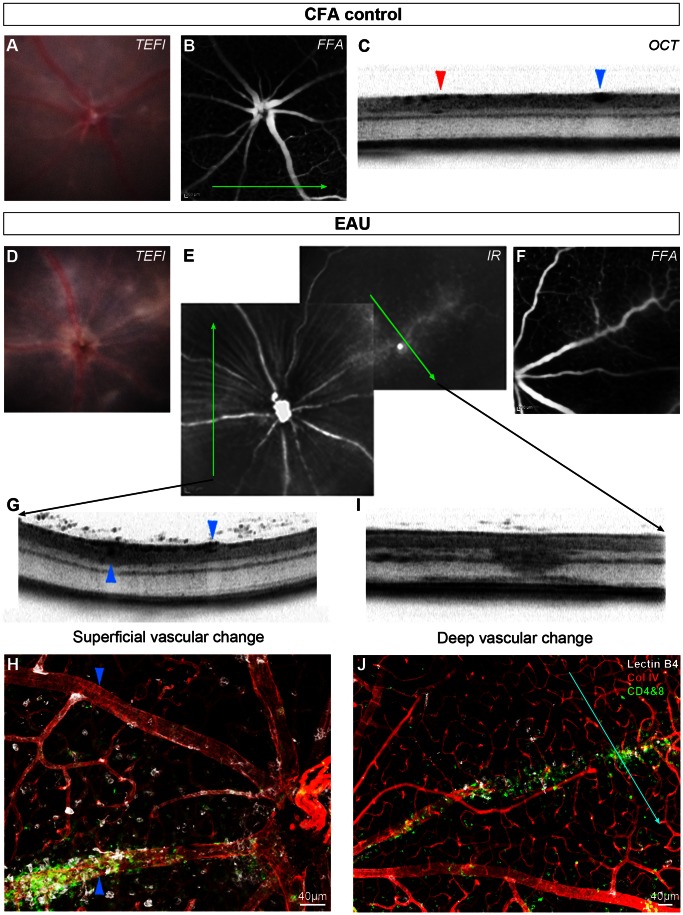
OCT identifies vascular changes in EAU and appearances are altered by depth and degree of infiltration. This CFA control eye has normal TEFI (**A**) and FFA (**B**) appearances. The green arrow indicates the orientation and position of the OCT scan (**C**), which transects a superficial retinal arteriole (red arrowhead) and vein (blue arrowhead). Matched images from an eye at day 36 post-induction of EAU demonstrate the range of changes (**D–J**). Corresponding disc swelling and vascular changes are seen on TEFI (**D**) and infrared SLO (**E**)**,** with mild leakage on FFA (**F**). Green arrows indicate the location and orientation of OCT scans. One scan transects two large vessels travelling near the retinal surface (**G**). The vessel (collagen IV in red) with altered OCT signal in the perivascular tissue (*upward pointing arrowheads*) is densely infiltrated with myeloid - lectin B4 binding (white) and T cell - CD4+ & CD8+ (green) cells as shown by immunohistochemistry on retinal flat mount (**H**). This is in contrast to the other vessel (*downward pointing arrowheads*), which lacks any localised infiltrate. Diffuse hyper-reflective signal on OCT (**I**) corresponds to a deep retinal vein, with associated cellular infiltrate (**J**). The blue line indicates alignment with the OCT scan.

Vasculitis or perivascular immune infiltrate is a predominant feature in murine EAU [1]. To assess vascular changes *in vivo* and *ex vivo*, we compared TEFI, FFA and OCT images of CFA control (**Figure 3A–C**) and EAU (**Figure 3D–G, I**) animals with corresponding immunohistochemistry for vascular and inflammatory cell markers on retinal flat-mounts (**Figure 3H & 3J**).

In CFA control mice, vessels assessed by TEFI (**Figure 3A**), FFA (**Figure 3B**) and OCT (**Figure 3C**), revealed a normal, non-inflamed appearance. In contrast in EAU, TEFI (**Figure 3D**) detects several abnormal, inflamed vessels that were also identified on corresponding infrared SLO (**Figure 3E**) and FFA (**Figure 3F**) images as well as on matched OCT scans (**Figure 3G & 3I**).

Two large superficial retinal vessels were observed on the OCT scan (**Figure 3G**) and their corresponding appearance assessed on flat-mount (**Figure 3H**). Interestingly, the abnormal vascular signal, enlargement and distortion of surrounding retinal tissue on OCT correlated to a dense perivascular infiltrate that surrounded the vessel (**Figure 3G** **,** upward pointing arrowhead) while the vessel lacking substantial perivascular accumulation of cells was small, dense and with preserved adjacent layers on OCT. (**Figure 3G** **,** downward pointing arrowhead).

In several mice, including wildtype, retinal vessels were occasionally observed below the inner nuclear layer. Inflammation of these vessels resulted in a dramatically different appearance on OCT (**Figure 3I**), with attenuation of signal in almost the full thickness of the retinal tissue and was often associated with evidence of fluorescein leakage in FFA (**Figure 3F**). Whilst it is not possible to detect oedema on retinal flat-mounts, myeloid and T-cells were found to be closely associated with these inflamed vessels (**Figure 3J**). Given that such changes can be detected on OCT, the origin and evolution of vasculitis, with its effects upon surrounding tissues might be further delineated *in vivo* using this technique.

### Detection of Novel Vascular Changes using OCT

**Figure 4:**
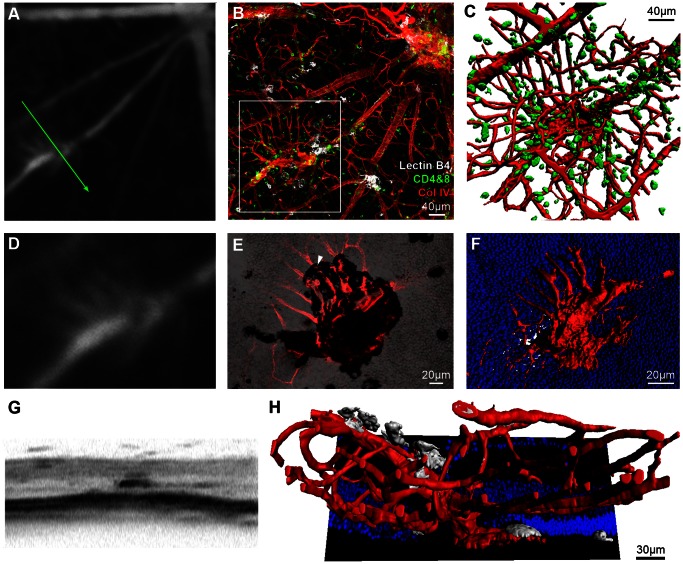
OCT imaging can guide identification and characterisation of novel features within the EAU model. Fundus fluorescein angiography (FFA) on an eye 36 days post-induction of EAU revealed an abnormality initially thought to lie within the pre-defined spectrum of vasculitis (**A**). Retinal flat-mount revealed an atypical vascular lesion arising along the course of a large vessel (**B**). 3D reconstruction of the region enclosed by the white box, with CD4+ & CD8+ cells (green) displayed (**C**). Magnified view of the FFA (**D**) correlates with a combined collagen IV (red) and brightfield projection image viewed from the subretinal space towards the vitreous (**E**). Note the black patch corresponding to RPE adherence during retinal separation. Vessels pass through this region (example indicated by white arrowhead) having arisen from the retina after penetrating the outer nuclear layer (nuclei in blue) - highlighted in the reconstruction (**F**). OCT demonstrates a unique, dense signal appearance (**G**), inconsistent with more widely observed vasculitic changes in EAU. The arrangement seen on sagittal reconstruction (**H**) might partly explain the appearance, which could be consistent with inflammatory induced intraretinal telangiectasia, vascular reorganisation or neovascular buds. Lectin B4 staining is displayed (white). The green arrow indicates the location and orientation of the OCT scan. See **Movie S2** for an animated 3D reconstruction.

Having established that key features known to occur in EAU can be identified, OCT was employed to describe vascular structures not classically recognised as part of the inflammatory process. During combined FFA and OCT imaging of mice from day 36 post-induction, an atypical pattern was detected and one representative example is presented (**Figure 4A**). The corresponding retinal flat-mount confirmed the lesion as vascular in origin and associated with both myeloid and T-cell infiltrates (**Figure 4B–C**). Detailed analysis of the tissue where separated from the RPE, revealed patches of pigment on brightfield microscopy, likely to reflect an area of abnormal retinal interaction with adjacent RPE (**Figure 4E**). Collagen IV labelled vessels appear to pass through this area as indicated. 3D reconstruction highlights vascular penetration through the outer nuclear layer and connection to the superficial retinal circulation on both sides of the plexus (**Figure 4F**). For a detailed view of the entire region, see **Movie S2**. The matched OCT scan shows an expansion of the layer corresponding to the RPE and an overall appearance that discriminates it from the early changes of vasculitis (**Figure 4G**). Using the reconstruction, it is possible to appreciate both how the OCT signal might be obtained and the association of activated myeloid cells (lectin B4 binding) with the vessels (Col IV, **Figure 4H**). Whether this lesion represents choroidal neovascularisation or intraretinal telangiectasia is uncertain, but it can be distinguished from typical vasculitis even with OCT alone.

### Derivation, Application and Calibration of the OCT Scoring System

Having ascertained that it is possible to detect core features during the development and course of EAU, we proceeded to design an OCT-based scoring system. Our focus was on end-user simplicity, speed and eliminating subjectivity. Scoring series acquisition for each eye involves eight OCT scans positioned around the posterior pole. Full details are described in **Figure S1**. Once established, the average imaging time was six minutes per eye.

Four features composed the scoring system, which were aligned to provide maximal correlation with histology and include a count of OCT signals in the vitreous (vitreous cellular infiltration), disc enlargement (oedema and cell infiltration), abnormal vasculature associated signals (vasculitis and perivascular infiltrate) and intraretinal (morphological) changes (**Table 1**). Vascular change, as a central feature of EAU was weighted accordingly. Vitreous opacities correlate to aggregates of infiltrating cells and can be counted objectively. The assessment of disc enlargement is a challenge, as on-machine measurement is strongly affected by many factors including hardware setup, focal plane and scan placement. The final measurement is an average of horizontal and vertical disc scans. In order to minimise false positive results, initial calibration against a test CFA control group is required (standardisation using the same protocol and OCT machine within each laboratory is necessary). All subsequent disc measurements are then determined as a ratio of the averaged CFA controls, which is squared and multiplied by a fixed weighting factor to combine with other scores (**Table 1**). Vascular change is expressed as a ratio of total scanned vessels against those demonstrating abnormal OCT signals. For intraretinal structural changes the peripheral scans are divided into ten equal segments and scored based on the number of segments with any abnormality. A worked example is provided in **Figure S4**.

**Table 1:** Range and calculation of final OCT based EAU scores.

Sub-scoringcategory	Description	Raw score range per eye	Alterationfactor	Maximum final EAU score
Vitreous involvement	Absolute count of discrete opacities in the vitreousspace up to a maximum of 150. The count from theworst affected scan is used.	0–150	None	0–150
Disc enlargement	On-machine measure of the linear distance between thetermination of the outer plexiform layer equivalent oneither side of the disc (See Figure 1). For each eye,average the results of the horizontal and verticaldisc scans.	Relative value to control CFA group	(Measure/average CFA control group measurement)^2^ then multiplied by 35	25.6–92.0(CFA control group average of 1695 units in this cohort)
Vascular involvement	Number of abnormal vessels as a ratio of total vesselsscanned based on OCT appearance. Two disccentred scans excluded.	0–1	Multiply by 300	0–300
Structural change	Using six scans (non-disc centred) only, each is dividedinto 10 equal vertical segments. The total number withany abnormality present is counted.	0–60	Multiply by 1.5	0–90
				**25.6–632**

Weighting was determined by correlation to histology, of which vascular change was most parallel. On average most scores will lie between 20 and 600 points. The range for the cohort in our experiments is presented.

### Scoring System Validation

**Figure 5:**
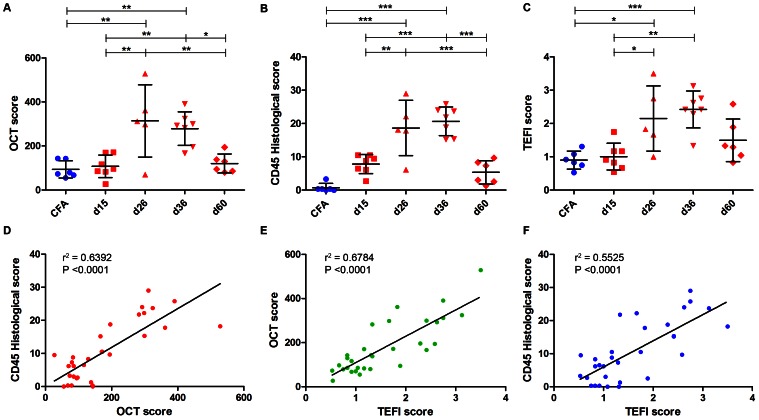
OCT-based EAU severity scoring correlates well with established measures and routine use should be considered. Absolute scores for the combined cohort at each time-point from day 15 to day 60 post-induction of EAU for OCT (**A**), histology (**B**) and TEFI (**C**). Each point shows the average of scores from three, masked, trained assessors. n = 31 eyes, treated independently by one-way ANOVA (P<0.0002 for each), with Tukey post hoc test for multiple comparison (* = P<0.05, ** = P<0.01, *** = P<0.001). SD are shown. Pearson correlations and linear regression of the values are displayed for comparisons between OCT and histological (**D**), TEFI and OCT (**E**) and TEFI and histological scores (**F**). Correlations are significant and correlate well between each other.

Whilst in humans, repeated Spectralis OCT scans can be locked to an identical location, as a result of optical constraints this process proves difficult in the mouse. Nevertheless, features can be manually followed in a single eye and to demonstrate the potential of OCT, examples are presented in **Figure S3**. Quantitative analysis is possible by combining data from all three cohorts to show the time-course of EAU scores generated using our OCT system, TEFI and CD45 immunolabelled histology (**Figure 5A–C**). Scoring was performed by three masked assessors and the averaged results are displayed. The data from each system are presented in raw form and not normalised to one scheme. All three modalities reflect the expected course of EAU and correlate significantly with each other (**Figure 5D–F**). OCT scores correlated well with histology (Pearson r^2^ = 0.6392, P<0.0001, n = 31 eyes), affirming the hypothesis that inflammatory changes in the limited 30-degree field of OCT are directly proportional to whole eye changes sampled by histology. OCT and TEFI correlate (r^2^ = 0.6784, P<0.0001), with TEFI also related to histology (r^2^ = 0.5525, P<0.0001).

## Discussion

In this study we have provided evidence that OCT imaging is a viable method for evaluating murine EAU and quantifying disease *in vivo.* We also present an OCT-based scoring system, which correlates well with existing TEFI and histology based analyses. OCT thus provides a tool for following dynamic intraretinal changes and will permit the reconciliation of mounting functional and cellular data from the EAU model.

### OCT Correlates with Current EAU Scoring Methods and has Distinct Advantages

In *C57BL/6* mice, for the range of pathology historically used to stage EAU severity – vitreous, disc, vessel and retinal structural changes – discrimination was possible. OCT can therefore be added to the range of techniques used for disease assessment, and will facilitate where appropriate, substitution for post-mortem histology and reduction and refinement of animal usage. Histology will inevitably still be performed for first describing novel findings and for corroborative immunophenotyping, but can otherwise be widely replaced by OCT, as it remains conceptually analogous and directly correlated to post-mortem histology scores. The overall time required for imaging and scoring is also markedly less per eye once processing, sectioning and staining for histology are accounted for.

A further advantage is that OCT can be used to directly observe features *in vivo* such as oedema or vascular leakage, which are not easily detectible on histology due to tissue processing. Furthermore, this platform has the ability to simultaneously perform FFA and scan at the exact location of any lesion. However, we noted that OCT alone was able to distinguish specific features in tissue surrounding vessels as a surrogate for leakage.

OCT delivers a closer substitute for histology than TEFI. Whilst current *in vivo* monitoring with TEFI can detect disc and vessel alterations, it cannot detect intraretinal or vitreous changes [10]. OCT avoids the disadvantages of intense light exposure, corneal contact and appears not to overestimate disc involvement to the same degree. Nevertheless, TEFI still has a role as a useful and quick screening step, identifying animals for subsequent detailed OCT scoring or other assessments.

Inter- and intra-animal variation in EAU models often result in a large spread and require many animals to obtain reproducible data from functional or cellular analysis. This data shows that OCT will allow intraretinal synchronisation of disease stage within experimental groups prior to sacrifice of animals, which consequently will improve outcome measures. By scoring disease without post-mortem histology, animals can be repurposed to other analyses (flow cytometry, Q-PCR, cytokine ELISA) with additional knowledge of the exact disease stage of each eye. Using OCT can also reduce overall animal numbers needed, consistent with universal targets for the replacement, reduction and refinement (3R’s) of animal use [21].

### OCT can Guide the Discovery and Characterisation of Novel Features

We have demonstrated that by employing OCT we can not only evaluate vitreous and intraretinal changes in more detail, but also identify and assess in a more targeted way, novel features of the inflamed retina. Using our platform, vessels can be imaged with combined FFA and OCT to localise leakage and analyse surrounding tissue in the associated areas. Deep retinal folds can be imaged and importantly, distinguished on OCT from other changes such as the abnormal vascular telangiectasia we identified. This feature in EAU was identified from day 36 onwards showing vascular remodelling to occur earlier in disease course than indicated by similar vascular membranes recently observed only from day 60 post-induction [22]. The vascular lesions share some similarities with those described in degenerative models with a chronic low grade inflammatory component, such as rhodopsin kinase knockout mice [23] or those carrying the *rd8* mutation [18]. The mice used in our study carry neither defect and thus the lesion may represent a more widespread feature of tissue remodelling in response to degenerative or inflammatory processes in the retina. Our data illustrates such changes, with vessels extending through the outer nuclear layer and under the RPE, before reconnecting with the superficial retinal vasculature. The vessels are unlikely to represent choroidal neovascularisation as neither FFA findings nor the irregular, yet ordered splay of the abnormal vessels are characteristic of this. Further analysis including 3D scanning electron microscopy to prove the integrity of Bruch’s membrane would be required to confirm this assertion.

### OCT Scoring and Future Directions

Construction of the OCT scoring system proved complex and retaining adequate capture of detail had to be balanced against user simplicity. Our system has been designed purposely to remove subjectivity where possible and unlike histological and TEFI scoring systems does not use imprecise stratification such as ‘mild, moderate or subtotal’. The structural sub-score, calibrated for broad overview and time efficiency does not distinguish between subtypes of retinal changes. As such it remains necessary to carefully study specific structural features as required for the precise purpose of each experiment.

As EAU in rats and *B10.RIII* mice is more severe [24], [25], the current data is based on *C57BL/6 *mice since it permits detailed imaging of the retina at critical stages. The largest constraint is the limitation of the degree of optical resolution. Further refinements in both the hardware and optics for rodent imaging will inevitability increase the quality of data obtained, which will allow progressively improved scoring accuracy and characterisation. Detailed analysis of choroidal changes may be possible using long wavelength OCT, whilst employing emerging adaptive optics technology may eventually allow the observation of dynamic processes on a single cell level *in vivo* [26]. More work to precisely delineate temporal changes and responses to therapeutic intervention with OCT are the likely next steps to be taken.

### Interpreting OCT Changes in Human Intraocular Inflammatory Disease by Histopathological Correlation with Murine EAU

Several of the features we have described in EAU reflect OCT changes seen in some human posterior segment inflammatory diseases [27], [28]. An additional major benefit of using our approach lies in the ability to directly correlate scan features with matched tissue, which can be directly analysed to identify immune cell composition and altered function. Due to the paucity of human material for histology, an identical human study has not been feasible. OCT is however, already being used in clinical practice to phenotype and monitor posterior uveitis [29]. By further examining other preclinical animal models, including humanised and spontaneous EAU in mice, more accurate correlations might be drawn [1]. Conversely, screening new EAU models with OCT for retinal changes closest to those of human disease, will allow selection and refinement during development. In the clinic, it remains challenging to judge remission of posterior segment inflammatory disease or detection of early recurrence and relapse. Quantification and analysis of OCT features other than macular oedema, as we have used here in EAU, could distinguish remission versus low-grade chronic inflammation as well as tissue remodelling in order to guide clinical decision-making.

## Supporting Information

Figure S1 **OCT based EAU scoring scan acquisition.** A summary of the imaging series for scoring, using an example composition. For each eye, eight combined IR+OCT scans are acquired **(A–H)**. Simultaneous FFA can be performed where required. The 30-degree field of view is centred on the optic nerve head. **A)** Horizontal scan through the disc. **B)** Horizontal scan, 30-degrees superior to the disc. **C)** Horizontal scan, 30-degrees inferior to the disc. **D)** Horizontal scan, 60-degrees superior to the disc. **E)** Vertical scan through the disc. **F)** Vertical scan, 30-degrees temporal to the disc. **G)** Vertical scan, 30-degrees nasal to the disc. **H)** Horizontal scan, 60-degrees inferior to the disc. See **Figure S4** for worked scoring of the same image set.
(TIF)

Figure S2 **The appearance and correlation with retinal anatomy obtained by OCT scanning.** An OCT scan from a non-induced *C57BL/6* mouse is displayed next to a matched histological section stained with haematoxylin and eosin.
(TIF)

Figure S3 **OCT can track the development of tissue changes over time.** Each column illustrates the development of a feature from the identical eye of an animal at the same manually located region on OCT for the four main features. Due to the intrinsic variability of the model, the timing and severity of changes varies between animals, but a general trend to early inflammation and late reduction in disease is evident. Note that it is impossible to directly quantify the degree of vitreous or intraretinal involvement using TEFI alone.
(TIF)

Figure S4 **A worked example of the scoring system using the image acquisition described in Figure S1.** Using all eight scans, each eye is scored across four domains and the raw results displayed. The number of discrete vitreous opacities, separated from the retina are counted in the scan that shows the strongest effect (**A**). Clusters that would receive scores are illustrated in the red box. A vertical and horizontal average of disc swelling is measured using on-machine software between the terminations of the layer corresponding to the outer plexiform layer (**B**). Across the six non-disc scans, the proportion of all abnormal vessels out of the total transected by all scans is calculated (**C**). The infrared scan can guide vessel location on the OCT (green arrow indicates position of the scan). Non-disc scans are then divided into ten equal vertical segments and the proportion containing any abnormality (including inflamed vessels, excluding vitreous changes) are counted (**D**). CFA control animal reference scans should be compared. We have not included discrete hyper-reflective changes along the outer plexiform layer or nerve fibre layer, as these are likely to reflect small vessels and appear in controls.
(TIF)

Movie S1 **3D reconstruction of retinal folds in an eye 80 days post-induction of EAU.**
(AVI)

Movie S2 **3D reconstruction of an intraretinal vascular abnormality in an eye 36 days post-induction of EAU.**
(AVI)
