# Supplementary figures and images for: Assessment and In Vivo Scoring of Murine Experimental Autoimmune Uveoretinitis Using Optical Coherence Tomography
Source: PLoS One. 2013 May 14;8(5):e63002. doi: 10.1371/journal.pone.0063002 (PMC3653962; doi:10.1371/journal.pone.0063002)

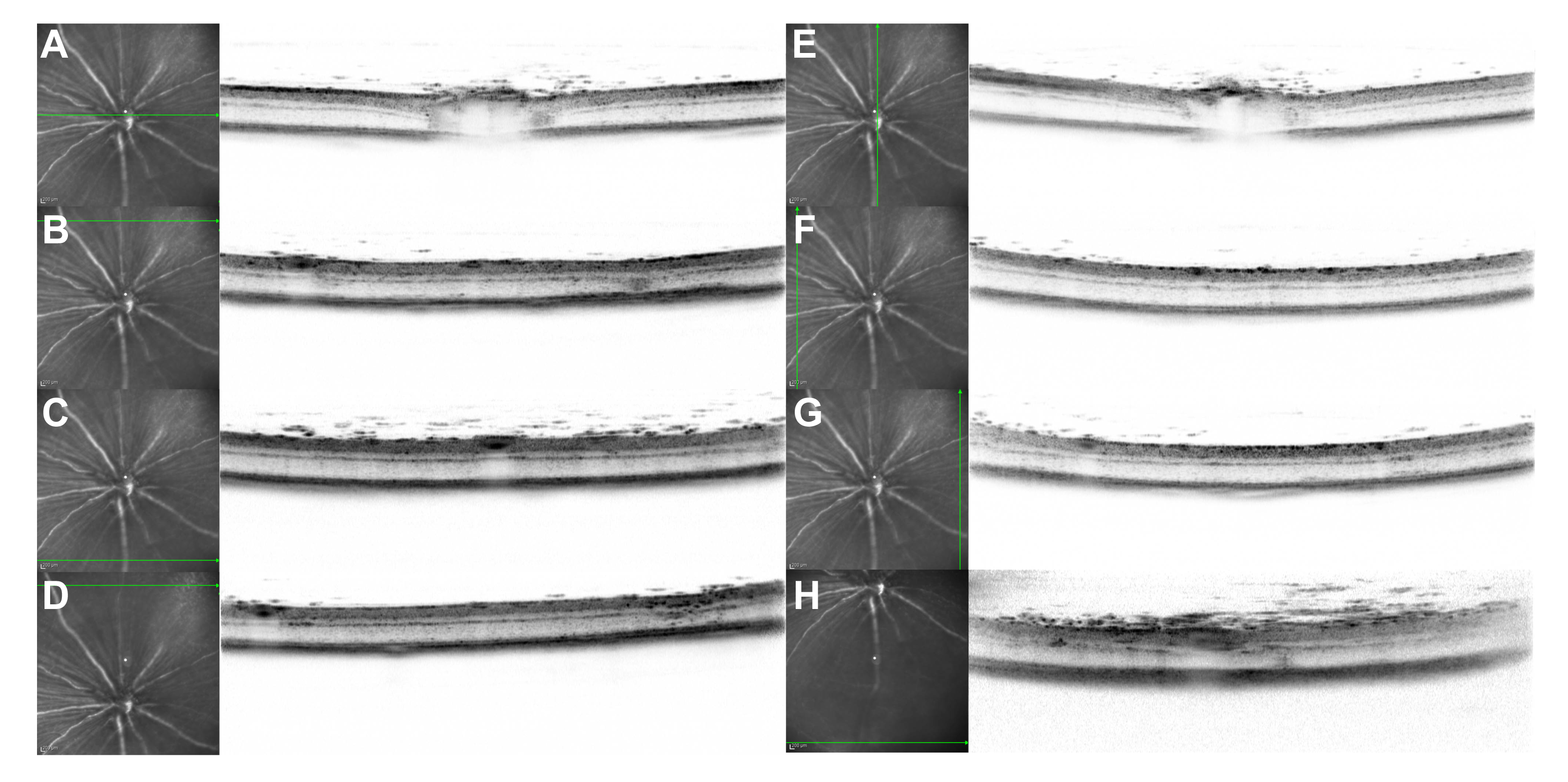

Supplement: Figure S1 — OCT based EAU scoring scan acquisition. A summary of the imaging series for scoring, using an example composition. For each eye, eight combined IR+OCT scans are acquired (A–H). Simultaneous FFA can be performed where required. The 30-degree field of view is centred on the optic nerve head. A) Horizontal scan through the disc. B) Horizontal scan, 30-degrees superior to the disc. C) Horizontal scan, 30-degrees inferior to the disc. D) Horizontal scan, 60-degrees superior to the disc. E) Vertical scan through the disc. F) Vertical scan, 30-degrees temporal to the disc. G) Vertical scan, 30-degrees nasal to the disc. H) Horizontal scan, 60-degrees inferior to the disc. See Figure S4 for worked scoring of the same image set. (TIF) [file pone.0063002.s001.tif]

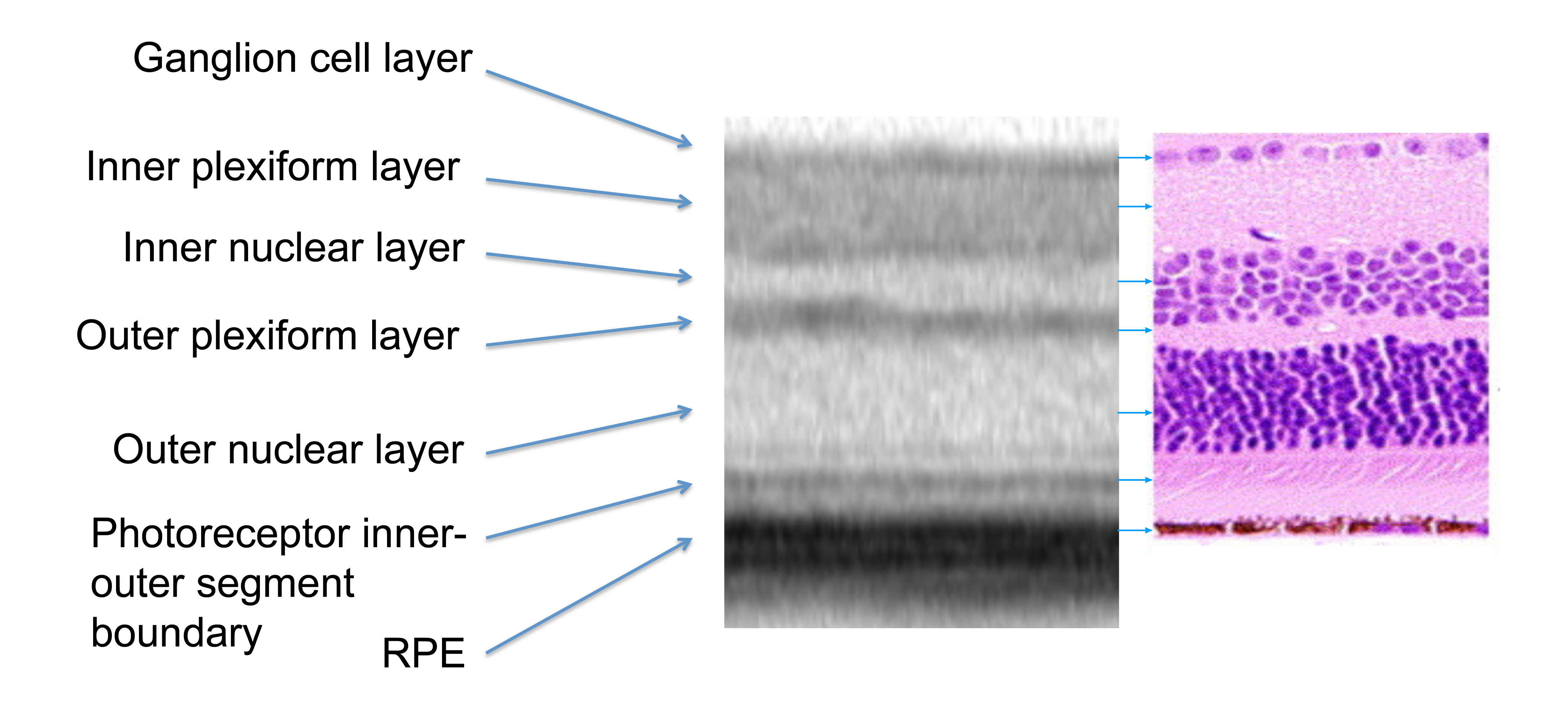

Supplement: Figure S2 — The appearance and correlation with retinal anatomy obtained by OCT scanning. An OCT scan from a non-induced C57BL/6 mouse is displayed next to a matched histological section stained with haematoxylin and eosin. (TIF) [file pone.0063002.s002.tif]

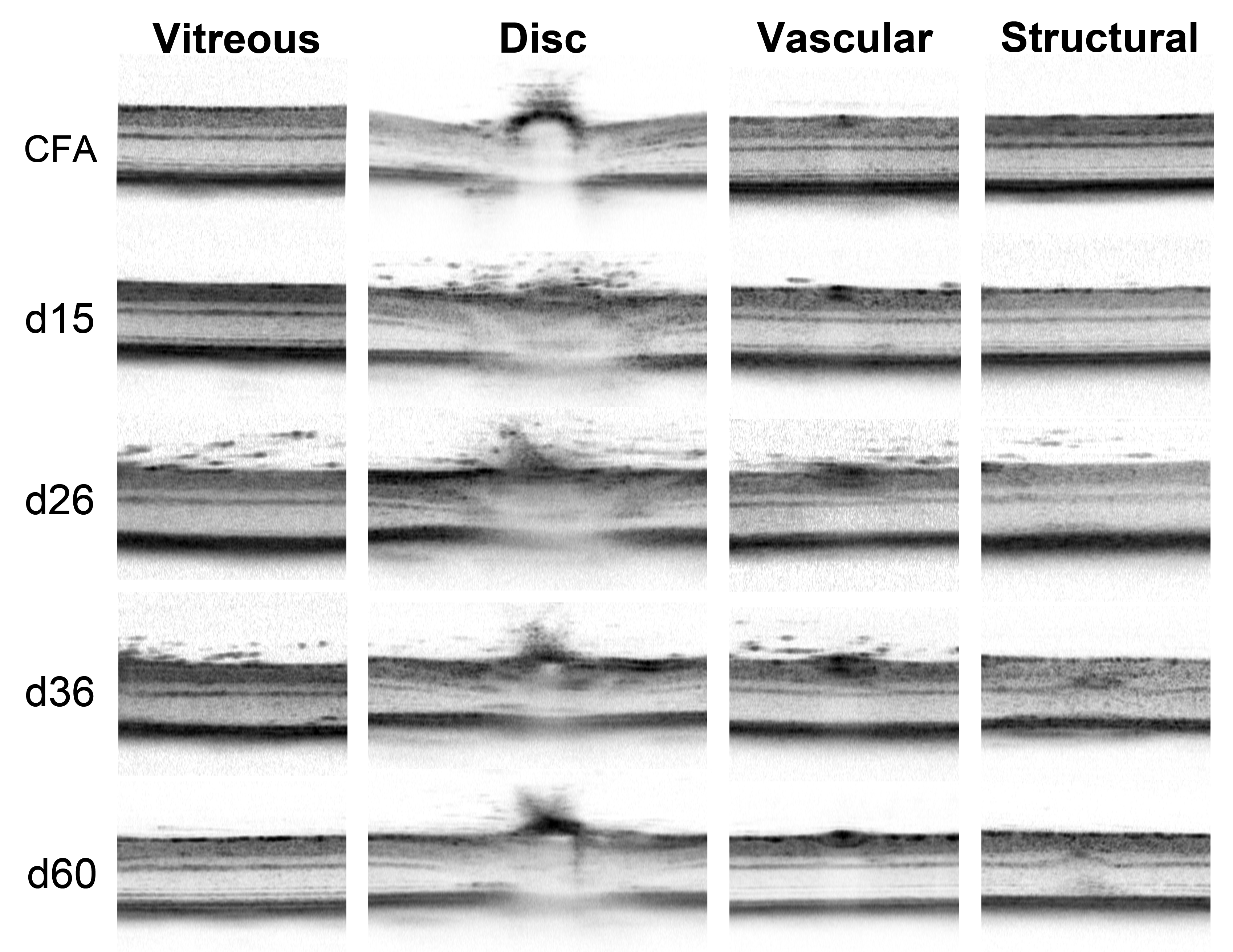

Supplement: Figure S3 — OCT can track the development of tissue changes over time. Each column illustrates the development of a feature from the identical eye of an animal at the same manually located region on OCT for the four main features. Due to the intrinsic variability of the model, the timing and severity of changes varies between animals, but a general trend to early inflammation and late reduction in disease is evident. Note that it is impossible to directly quantify the degree of vitreous or intraretinal involvement using TEFI alone. (TIF) [file pone.0063002.s003.tif]

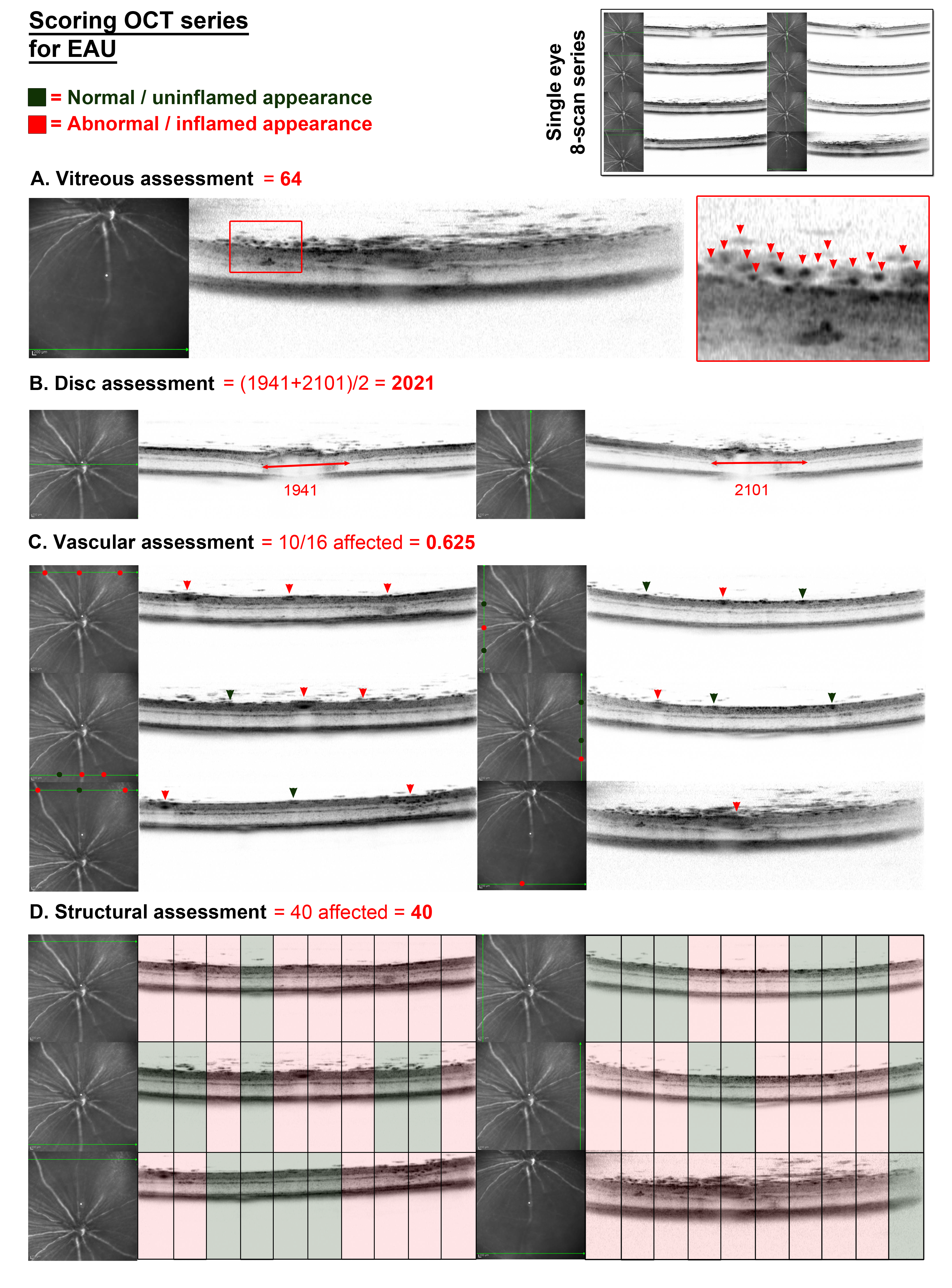

Supplement: Figure S4 — A worked example of the scoring system using the image acquisition described in Figure S1. Using all eight scans, each eye is scored across four domains and the raw results displayed. The number of discrete vitreous opacities, separated from the retina are counted in the scan that shows the strongest effect (A). Clusters that would receive scores are illustrated in the red box. A vertical and horizontal average of disc swelling is measured using on-machine software between the terminations of the layer corresponding to the outer plexiform layer (B). Across the six non-disc scans, the proportion of all abnormal vessels out of the total transected by all scans is calculated (C). The infrared scan can guide vessel location on the OCT (green arrow indicates position of the scan). Non-disc scans are then divided into ten equal vertical segments and the proportion containing any abnormality (including inflamed vessels, excluding vitreous changes) are counted (D). CFA control animal reference scans should be compared. We have not included discrete hyper-reflective changes along the outer plexiform layer or nerve fibre layer, as these are likely to reflect small vessels and appear in controls. (TIF) [file pone.0063002.s004.tif]
